# Draft Genome Sequence of *Sphingobium yanoikuyae* TJ, a Halotolerant Di-*n*-Butyl-Phthalate-Degrading Bacterium

**DOI:** 10.1128/genomeA.00569-16

**Published:** 2016-06-16

**Authors:** Decai Jin, Ying Zhu, Xinxin Wang, Xiao Kong, Huijun Liu, Yafeng Wang, Ye Deng, Minghong Jia

**Affiliations:** a CAS Key Laboratory of Environmental Biotechnology, Research Center for Eco-Environmental Sciences, Chinese Academy of Sciences, Beijing, China; b Institute of Biology, Gansu Academy of Sciences, Lanzhou, China; c China Offshore Environmental Service Co. Ltd., Tianjin, China; d Beijing Key Laboratory of Detection and Control of Spoilage Organisms and Pesticide Residues in Agricultural Products, Beijing University of Agriculture, Beijing, China

## Abstract

*Sphingobium yanoikuyae* TJ is a halotolerant di-*n*-butyl-phthalate-degrading bacterium, isolated from the Haihe estuary in Bohai Bay, Tianjin, China. Here, we report the 5.1-Mb draft genome sequence of this strain, which will provide insights into the diversity of *Sphingobium* spp. and the mechanism of phthalate ester degradation in the estuary.

## GENOME ANNOUNCEMENT

Members of the genus *Sphingobium* are widely distributed in nature and play an important role in biodegradation and bioremediation (1). *Sphingobium yanoikuyae* TJ was isolated from a seawater sample collected from the Haihe estuary, Tianjin, China. It was assigned to the *Sphingobium* genus based on its 16S rRNA sequence. *S. yanoikuyae* TJ could degrade di-*n*-butyl phthalate (DBP), one of the most used phthalate esters as a sole carbon and energy source and a pollutant of concern in various environments. The characteristics of *S. yanoikuyae* TJ for biodegradation of DBP were reported in our previous study (2). So far, the genome sequences of several *Sphingobium* spp., such as *Sphingobium* sp. strain SYK-6 (3), *S. yanoikuyae* strain B1 (4), *S. yanoikuyae* strain XLDN2-5 (5), *Sphingobium* sp. strain YL23 (6), *Sphingobium* sp. strain C100 (7), and *Sphingobium* sp. strain ba1 (8), have been published. However, a genome sequence for the highly efficient degradation of DBP by a *Sphingobium* species has not been reported.

The genomic DNA of *S. yanoikuyae* TJ was sequenced using the Illumina MiSeq platform at the Major BioTech Co. Ltd. (Shanghai, China). A total of 831.4 Mb of paired-end reads, with an average insert size of 300 bp, were produced, providing approximately 187-fold coverage. Filtered reads were assembled, scaffolded, and gap-filled by SOAPdenovo version 2.04 (9) and GapFiller version 1.10 (10). The final assembly contains 124 contigs—with the largest length being 344,118 bp—which were assembled into 105 scaffolds with an *N*_50_ length of 137,832 bp. The genome sequence was annotated using the NCBI Prokaryotic Genome Annotation Pipeline (PGAP) (http://www.ncbi.nlm.nih.gov/genome/annotation_prok).

The draft genome of *S. yanoikuyae* TJ consists of 5.1 Mb with a G+C content of 64.4%. A total of 4,606 coding sequences (CDSs), 102 pseudogenes, 3 noncoding RNAs (ncRNAs), 50 tRNAs, and 3 rRNA operons were identified. Of the CDSs, 85.9% were assigned to clusters of orthologous groups, with amino acid transport and metabolism being the most abundant class. An average nucleotide identity analysis revealed that *S. yanoikuyae* TJ is phylogenetically related to *S. yanoikuyae* ATCC 51230 (95.5%).

In particular, we analyzed the genes that are possibly responsible for the degradation of phthalate esters (PAEs). A gene-encoding serine hydrolase (AYR46_23250) shared 99% identity with the esterase gene (GenBank accession no. AJO67803) of *Sphingobium* sp. SM42, which is responsible for initial PAE decomposition. Moreover, the *ophA1*, *ophB*, and *ophC* genes (AYR46_23140, AYR46_23145, and AYR46_23150), which are responsible for *o*-phthalate degradation and the encoding oxygenase components of phthalate 4,5-dioxygense, 4,5-dihydroxyphthalate dehydrogenase, and 4,5-dihydroxyphthalate decarboxylase, respectively, were found in the draft genome of strain TJ. However, no proteins showed similarity to *ophA2* (reductase component of phthalate 4,5-dioxygense), and the absence of this gene might be the reason why strain TJ cannot utilize phthalic acid, which is the main intermediate metabolite during the degradation of PAEs. In addition, one betaine-aldehyde dehydrogenase gene and one liter-ectoine synthase gene, which are important for survival in a saline estuary, were identified. The genome sequence of *S. yanoikuyae* TJ and its annotation will provide further insight into the PAEs degradation mechanism of *Sphingobium* spp.

### Nucleotide sequence accession numbers.

This whole-genome shotgun project has been deposited at DDBJ/ENA/GenBank under the accession number LSVG00000000. The version described in this paper is the first version, LSVG01000000.
